# Characterization and Pathological Analysis of a Virulent *Edwardsiella anguillarum* Strain Isolated From Nile Tilapia (*Oreochromis niloticus*) in Korea

**DOI:** 10.3389/fvets.2020.00014

**Published:** 2020-01-28

**Authors:** Woo Taek Oh, Jin Woo Jun, Hyoun Joong Kim, Sib Sankar Giri, Saekil Yun, Sang Guen Kim, Sang Wha Kim, Jeong Woo Kang, Se Jin Han, Jun Kwon, Se Chang Park

**Affiliations:** ^1^Laboratory of Aquatic Biomedicine, College of Veterinary Medicine and Research Institute for Veterinary Science, Seoul National University, Seoul, South Korea; ^2^Department of Aquaculture, Korea National College of Agriculture and Fisheries, Jeonju, South Korea

**Keywords:** nile tilapia, Edwardsiellosis, Korea, *Edwardsiella anguillarum*, aquaculture

## Abstract

*Edwardsiella* species are one of the top causative pathogens of mortality in various fisheries worldwide. Their role in zoonotic infections and increase in antibiotic-resistance has raised concerns and interests in many research fields. Similar to the studies investigating human clinical cases, there has been an increase in research examining the potential pathogenic role of the bacterium in aquaculture. Within the *Edwardsiella* family, *Edwardsiella anguillarum* was lastest group to be differentiated from the *Edwardsiella tarda* group, and many studies focusing on the virulence of this species have since ensued. In Korea, only *E. tarda* infections have been reported in aquaculture industries, and there have been no reports on economic losses incurred owing to *E. anguillarum* infection. There has been a recent report investigating the pathogenicity and pathological changes caused by *E. anguillarum* infection in a tilapia farm located in the Costa Rica. To the best of our knowledge, as ours is the first report of *E. anguillarum* infection in a Nile tilapia (*Oreochromis niloticus*) farm located in an Asian country, the pathogenicity of the bacterial strain was histopathologically compared to that of the past studies. As tilapia is one of the most globally consumed fish species, particularly throughout Asia, Europe, and America, an epidemiological study regarding the disease distribution is necessary for the control and prevention of the disease. Here, we report the first mass mortality case caused by *E. anguillarum* infection in a Nile tilapia farm located in Korea; the bacterial strain responsible was isolated, characterized, and pathologically analyzed.

## Introduction

*Edwardsiella* is one of the serious pathogenic bacteria affecting global aquaculture in various cultured fish species (1). The genus *Edwardsiella* comprise rod-shaped, gram-negative, facultative anaerobic bacteria belonging to the family *Enterobacteriaceae* and order Enterobacteriales (2). The motility of these bacteria depends on the existence of flagella; while *Edwardsiella* is largely considered motile, there have been reports of some bacteria being non-motile due to the deletion of the flagella biosynthetic gene (3). The genus *Edwardsiella* comprises five species: *Edwardsiella hoshinae, Edwardsiella ictaluri, Edwardsiella tarda, Edwardsiella piscicida*, and *Edwardsiella anguillarum* (4); *E. tarda* is one of the most common species found under normal environmental conditions (5), and infects a variety of animals including fish, amphibians, and mammals (6, 7). Due to its transmission as a zoonotic pathogen, and the opportunistic spread of infection in hospital patients, these bacteria have received attention regarding its control and treatment options for infection (6, 8). Three species of *Edwardsiella* are currently considered the leading aquatic pathogens in global aquaculture (9); the first is *E. ictaluri*, a non-motile species responsible for mass mortality caused by septicemia in catfish (10); the second is *E. piscicida*, which is reported as a typical motile fish pathogen causing mortality in turbot (*Psetta maxima*) (11), striped bass (*Morone saxatilis*) (12), and eels (*Anguilla japonica*) (13); and third, similar to *E. piscicida, E. anguillarum* was distinguished from *E. tarda* as an atypical non-motile fish pathogen *tarda* (4), and isolated from various aquatic species including groupers (*Epinephelus* spp.), seabream species, channel catfish (*Ictalurus punctatus*), hybrid catfish (*Ictalurus furcatus*), and tilapia (*Oreochromis* sp.) (3, 14–16).

*Edwardsiella piscicida* and *E. anguillarum* species were originally included in the *E. tarda* group, but because of improvements in complete sequencing technology and the identification methods of the bacteria, the aforementioned species were divided into new clades (4, 17). However, the analysis of phenotypic characteristics varied between the strains and the criteria differentiating the groups remained ambiguous (18); as such, more definitive methods for the identification of the bacteria was developed by using species-specific polymerase chain reaction (PCR) primers (19). In addition, phylogenetic analysis of the highly conserved 16S ribosomal RNA (rRNA) and several housekeeping genes revealed distinct features of each group, resolving independent groups according to species (20). Using these techniques, various strains formerly identified as *E. tarda*, were re-grouped into *E. piscicida* and *E. anguillarum*.

*Edwardsiella anguillarum* shares similar characteristics to other *Edwardsiella* isolates, such as the growth capability under anaerobic conditions; however, its non-motile nature differentiated it from other groups (21). These bacteria were originally isolated from diseased eels (*Anguilla* spp.) in China, and studies concerning the virulence of the bacterial isolates from a variety of fish species have been increasing in other countries, including China, Japan, USA, and Greece (3, 4, 15). In this study, we aim to identify the species of *Edwardsiella* isolated from infected Nile tilapia cultured in Korea; we additionally attempt to characterize the pathogenicity of the species identified, examine the damage to internal organs by histopathological examination, and determine antimicrobial susceptibility.

## Materials and Methods

### Outbreak of the Disease

The disease outbreak occurred in April 2019, on a tilapia farm located in the Chungbuk Province of Korea. According to the farm owner, mortality rates were low under typical conditions on this farm, as tilapia is one of the most environmentally adaptable and disease-resistant fish species in aquaculture (22). An increase in mortality was observed over a month on the farm, and the cumulative mortality was estimated at 20% during the 2 weeks of observation. The farm reared a total of 50,000 tilapia, distributed between 6 water tanks filled with 15 tons of water each that were maintained at 30°C for optimum growth and 6.6 mg/L of dissolved oxygen. The facility comprised a half closed re-circulating system and a half open circulating system, which were supplemented with ground water. Ten diseased tilapia were sent to the Laboratory of Aquatic Biomedicine at Seoul National University, Korea, for a diagnostic evaluation of the disease-causing agent. The diseased fish were examined visually and microscopically for any external lesions. Every fin and gill was swabbed and smeared onto glass slides for light microscope observations. For the examination of gross changes, the spleen, liver, and kidneys of the fish were removed and visually inspected, before further post-mortem examinations were performed.

### Post-mortem Examination and Bacterial Isolation

Owing to the low and continuous mortality rate, the causative agent of the disease was considered to be bacterial rather than viral or parasitic. To test for parasite infection, the gills of the fish were swabbed and smeared on glass slides and observed under a light microscope. To test for viral infection, specific diagnostic primers for Tilapia lake virus (TiLV) were used to screen for the virus (23), which was recently observed to be spreading sporadically throughout many countries. To test for bacterial infection, the internal organs, including the kidneys, liver, and spleen, were collected and individually streaked onto Tryptic Soy Agar (TSA) (BD Difco, Sparks, MD, USA) supplemented with 5% sheep blood; the plates were incubated at 30°C under aerobic conditions and examined every 12 h for 3 d. Representative colonies, which covered the largest percentage of the dish compared to other colonies, were selected and sub-cultured for pure isolation. The sub-cultured bacteria was grown on TSA at 30°C, and finally stored at −80°C in 30% glycerol until further use.

### Biochemical Characterization of the Isolate

The isolated bacterial strain was cultured on TSA at 30°C for 24 h before being phenotypically characterized. The morphology of the bacteria was examined using a Gram-staining kit (bioMérieux, Marcy-I'Etoile, France). The catalase activity response was examined in the presence of 3% (v/v) aqueous hydrogen peroxide solution, and the oxidase activity using 1% tetramethyl p-phenylenediamine (Merck, NJ, USA). Bacterial motility was examined by cultivating the bacteria in Tryptic Soy Broth (TSB) (BD Difco, Sparks, MD, USA) and observing potential movement under a light microscope. The growth test under various temperatures and NaCl concentrations entailed growing the strain in TSB for 48 h under temperatures ranging from 4 to 40°C, and at 0, 2, 4, 6, 8, and 10% concentrations of TSB. The biochemical characteristics of the strain were examined by testing the media with API 20E and API 50CH (bioMerieux, France) rapid diagnostic kits, according to the manufacturer's protocol.

### Identification of the Bacteria and Phylogenetic Analysis

For the identification of the bacteria, the total DNA was extracted from the bacteria of freshly grown culture plates using a DNeasy blood and tissue kit (Qiagen, CA, USA) following the manufacturer's instructions. PCR was performed using the extracted DNA; the specific diagnostic primers and assay conditions used were the same as previously described (19). For the identification of bacterial subspecies, 16S rRNA and 7 housekeeping genes [*phoR* (phosphate regulon sensor protein), *adk* (adenylate kinase), *aroE2* (shikimate 5-dehydrogenase), *metG* (methionyl-tRNA synthetase), *mdh* (malate/lactate dehydrogenase), *dnaK* (molecular chaperone), and *pyrG* (CTP synthetase)] were sequenced by PCR using specific primers targeting the individual genes under the same conditions as those described by Frank et al. (24) Abayneh et al. (17). For gene sequence analysis, PCR products were sent to the genomic division of Macrogen (Korea), where nucleotide sequence analysis was performed using the ABI PRISM 3730XL Analyzer, with BigDye^®^ Terminator v3.1 Cycle Sequencing Kits (Applied Biosystems, USA). The 16S rRNA gene sequence was compared to other bacteria species using BLASTn software from the National Center for Biotechnology Information (NCBI, Bestseda, MD) and EzBioCloud server (https://www.ezbiocloud.net/). The sequences of the 7 housekeeping genes were collected and aligned, using BioEdit software, with other bacterial strains registered in the GenBank server (GenBank accession numbers: MN225931, MN225932, MN225933, MN225934, MN225935, MN225936, and MN218777). The concatenated phylogenetic tree, comprised of the combined sequences of the 16S rRNA and 7 housekeeping genes, was constructed using MEGA 7.0 software; a bootstrap analysis was performed with 1,000 replications, and the phylogenetic tree was constructed using the neighbor-joining method (25).

### Challenge Trial and Pathogenicity Analysis

To demonstrate the pathogenicity of the bacterial strain, Nile tilapia, of an average weight of 20 g, were purchased from another tilapia farm located in the Jeonbuk Province, Korea. The trial was prepared on triplicate form with 10 fish per each group in 100L fresh water. The trial followed ethical guideline of Department of Aquaculture, Korea National College of Agriculture and Fisheries strictly on handling fish. The bacteria were grown and prepared in TSB, incubated at 30°C with shaking, under aerobic conditions. The concentration of bacteria was calculated based on the optical density measured using a SmartSpec^TM^ 3000 spectrophotometer (Bio-Rad, CA, USA). The titration was performed by diluting with Phosphate Buffered Saline (PBS), and the bacteria were centrifuged at 8000 × *g* immediately before the inoculation and washed using PBS. The titration of the bacteria was calculated by the number of cells suspended in 0.1 mL PBS at 3 × 10^7^, 10^6^, 10^5^, 10^4^, and 10^3^ CFU per fish, and the same volume of PBS was injected intraperitoneally for the control groups. All infection experiments were simultaneously performed in triplicate, and the challenge fish in each group were maintained in separate 120 L water tanks. Each group consisted of ten fish, including the control group, and the water temperature was maintained at 30°C using titanium heaters. The challenge trials were performed over 15 d, and fish exhibiting severe clinical symptoms of infection were collected immediately after death for histopathological examination. The remaining fish were observed for the residual time to calculate the survival rate during the test. After the end of the trial, the dead fish were collected and used for the re-isolation of the bacteria. The re-isolation procedure was performed using the same conditions described in the prior bacterial isolation step.

### Histology Examination

The fish exhibiting severe clinical signs were collected immediately after death; these fish showed typical signs of bacterial infection, such as imbalanced or lethargic activity and erratic swimming motions, with a few instances of proptosis observed. The internal organs, including kidneys, liver, and spleen, were separately excised from the fish and collected for examination. The tissues were preserved in 10% neutral-buffered formalin, and following fixation, the tissues were trimmed, dehydrated using ethanol, and embedded in paraffin blocks, which were sectioned and stained with hematoxylin and eosin for the analysis. The specimens were examined using light microscopy and digitally scanned by Xenos Inc. (Korea).

## Results and Discussion

### Post-mortem Examination of Diseased Fish

Upon visual inspection of the diseased fish, there were no significant external lesions that are commonly observed, but proptosis was evident, which is a typical clinical symptom of bacterial infection. Upon internal organ examination, signs of congestion and necrosis of the liver were clearly observed, with no infection-related symptoms observed in the spleen and kidneys of the diseased fish. In the parasite detection test, the glass slides smeared with gill-swabs showed a negative result under microscope observation. In the viral infection examination, the PCR result showed clear negative results, which were as expected considering the mortality rate and contagion rate was relatively slower than any viral infections. In the bacterial isolation experiment, off-white colonies, with a diameter of ~1–2 mm, were observed on the plates of TSA agar supplemented with sheep blood after 24 h incubation at 30°C. The dominant colonies, with over 90% coverage and showing uniform shape and color, were selected for further analysis and stored in a glycerol solution.

### Identification of the Bacteria

The phenotypic characterization of the strain was performed using API 20E and API 50CH (bioMerieux, France); however, some ambiguous responses and contraindicative patterns were observed between species (Table 1). Referring to past study results, the *E. anguillarum* strain SNU2 which was isolated in this study, was unable to ferment inositol, sorbitol, rhamnose, saccharose, melibiose, amygdalin, lactose, and trehalose like other *Edwardsiella* strains (4). Additionally, the production of β-galactosidase, arginine dihydrolase, urease, tryptophan deaminase, and gelatinase were also negative, similar to other *Edwardsiella* species. The response for glucose and D-galactose acidification were positive like other *Edwardsiella* strains, but the SNU2 strain showed a different response to *E. anguillarum* strain ET080813 in mannitol fermentation, arabinose fermentation, and response to esculine (4). Also, H_2_S production and glycerol fermentation were contraindicative patterns to *E. tarda* strain NCTC 10396 (20). Another Edwardsiella isolate from diseased catfish cultured in Korea which was isolated as *E. tarda* previously and re-classified into E. piscicida group (20) showed one different phenotypic characteristic in Esculine. The morphology test result was same as other *Edwardsiella*, which was gram-negative, oxidase-negative, and catalase-positive. The strain was non-motile like other *E. anguillarum* species and could tolerate growth temperatures of up to 40°C and an NaCl concentration of 4%. Strain SNU2 which was isolated from one of the tilapia farm located in Chungbuk province in Korea showed similar phenotypical characteristics to other *E. anguillarum* strains isolated from other countries, but, as expected from past research indicating that *Edwardsiella* species cannot be distinguished by its biochemical details alone (18), there were only a few responses different to other strains.

**Table 1:** API 50CH and API 20E test result done for the phenotypic characterization of strain SNU2 compared to other *Edwardsiella* species type strains and *E. tarda* strain isolated from diseased catfish cultured in Korea.

	***E. anguillarum* SNU2 (this study)**	***E. anguillarum* ET080813T**	***E. piscicida* ET883T**	***E. tarda* NCTC 10397T**	***E. tarda* ETK01**
Insoitol fermentation	–	-	–	–	–
Sorbitol	–	–	–	–	–
Rhamnose	–	–	–	–	–
Saccharose	–	–	–	–	NA
Melibiose	–	–	–	–	–
Amygdalin	–	–	–	–	–
Lactose	–	–	–	–	NA
Trehalose	–	–	–	–	NA
β-galactosidase	–	–	–	–	–
Arginine dihydrolase	–	–	–	–	–
Urease	–	–	–	–	–
Tryptophan deaminase	–	–	–	–	–
Gelatinase	–	–	–	–	–
Glucose acidification	+	+	+	+	+
D–galactose acidification	+	+	+	+	NA
Mannitol fermentation	–	+	–	–	–
Arabinose fermentation	–	+	–	–	–
Esculine	+	–	–	–	–

*+, positive; −, negative; NA, Not Available*.

### Phylogenetic and Sequence Analysis

**Figure 1:**
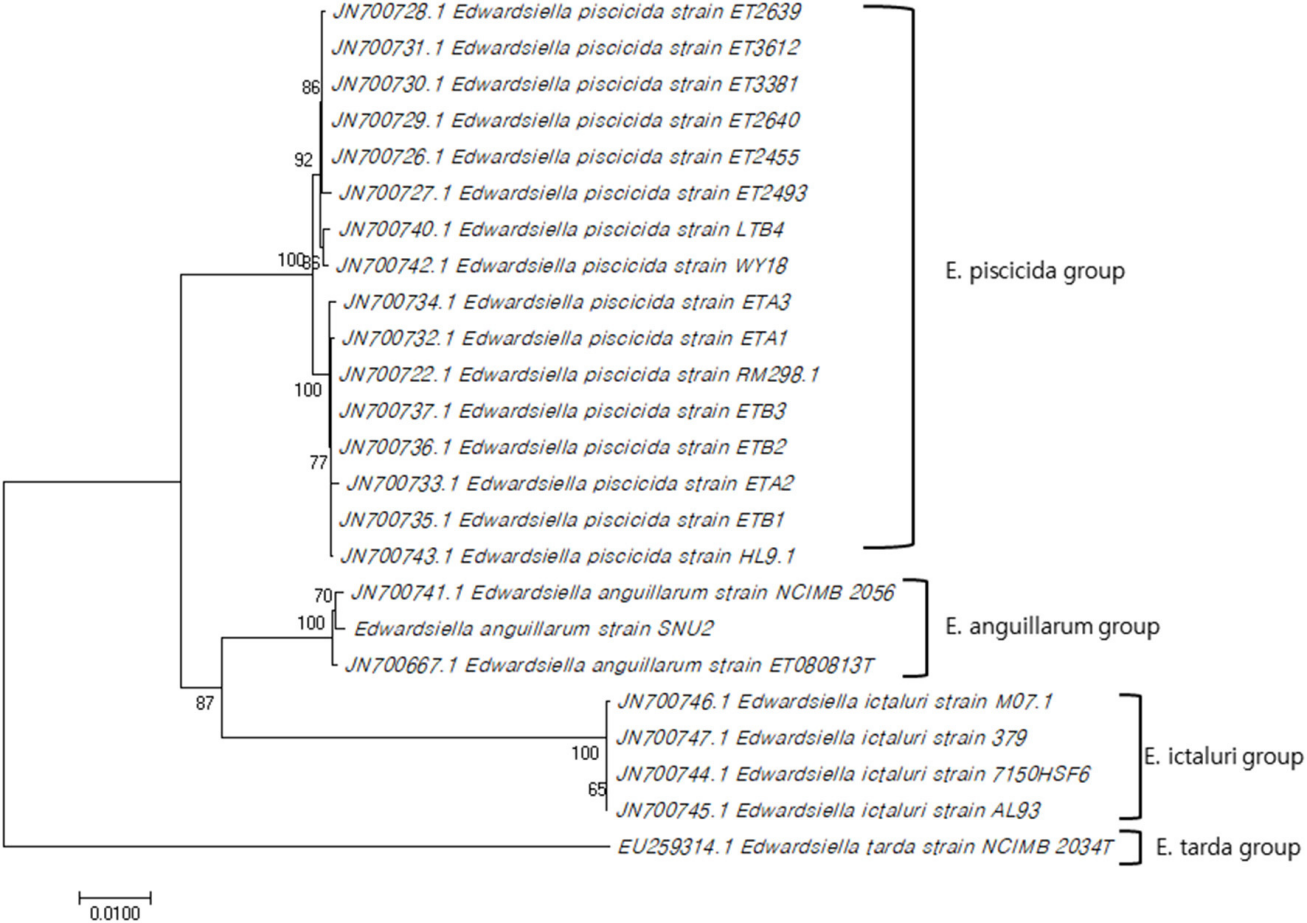
Concatenated phylogenetic tree using 16S rRNA gene sequence and seven house-keeping genes by MEGA 7.0 software using neighbor-joining method and the bootstrap analysis was performed with 1000 replicates (Genbank accession numbers used for this study: MN203720, MN225931, MN225932, MN225933, MN225934, MN225935, MN225936, MN218777).

The 16S rRNA gene sequence of strain SNU2 (GenBank accession number: MN203720) showed the highest similarity to *E. anguillarum* strain ET080813T and *E. ictaluri* strain ATCC 33202T (both 99.86%), followed by the *E. tarda* strain NBRC 105688T (99.45%), and the *E. hoshinae* strain NBRC 105699T (99.45%). Additionally the 16S rRNA gene sequence of strain SNU2 shared 99.71% similarity to that of strain ETK01 which is the only isolate reported as fish pathogenic *E. tarda* in Korea. The phylogenetic tree constructed using the 16S rRNA and 7 housekeeping gene sequences (GenBank accession numbers: MN225931, MN225932, MN225933, MN225934, MN225935, MN225936, and MN218777) showed that strain SNU2 was included in the clade of *E. anguillarum* (Figure 1) and closest *to E. anguillarum* strain NCIMB 2056, as well as being clearly differentiated from *E. piscicida* and *E. ictaluri*. In addition, the PCR, using specific diagnostic primers for *E. anguillarum*, showed positive results for the strain and the phylogenetic tree confirmed the strain to be clearly included in the *E. anguillarum* clade, along with other strains isolated from Sharpsnout seabream, eels, and groupers. Considering the results of the diagnostic PCR, phylogenetic tree, and biochemical characterization, strain SNU2 was concluded to be of the *E. anguillarum* species, which is the first time that *E. anguillarum* infection has been reported in Korean aquaculture.

### Pathogenicity Challenge Trial

**Figure 2:**
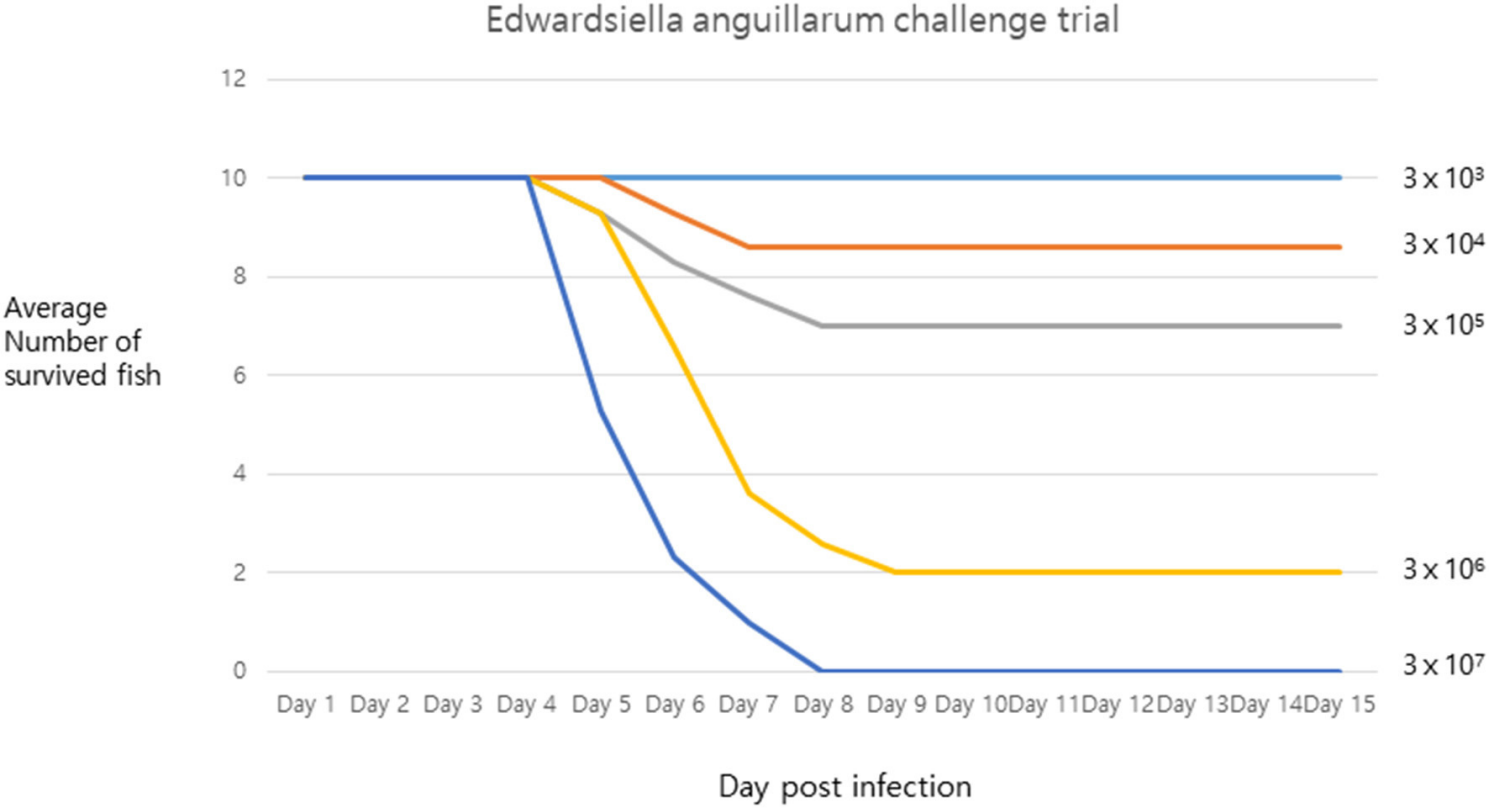
The challenge trial result describing survival rate of infected fish under x axis representing days post infection and y axis representing average number of survived fish.

The pathogenicity of strain SNU2 was shown to cause the same level of infection as that observed in the tilapia farm, in the same fish species living under similar conditions. Results showed that death occurred 4 days post-infection, and the lethal dose 50 (LD_50_) was found to be between 3 × 10^6^ and 3 × 10^5^ (Figure 2). The survival rate of the fish challenged over the titer 3 × 10^7^ was zero percent. A recent report showed that *E. anguillarum* infection in Sharpsnout seabream had an LD_50_ of 1.85 × 10^4^ at 48 h (3); compared to this study, strain SNU2 was less virulent and the contagion rate was slower; this discrepancy might be explained, in part, by the fact that the two studies were performed in different fish species, i.e., the bacterial strain in the study by Katharios et al. (3) was isolated from Sharpsnout seabream and tested in zebrafish.

### Histopathological Analysis

**Figure 3:**
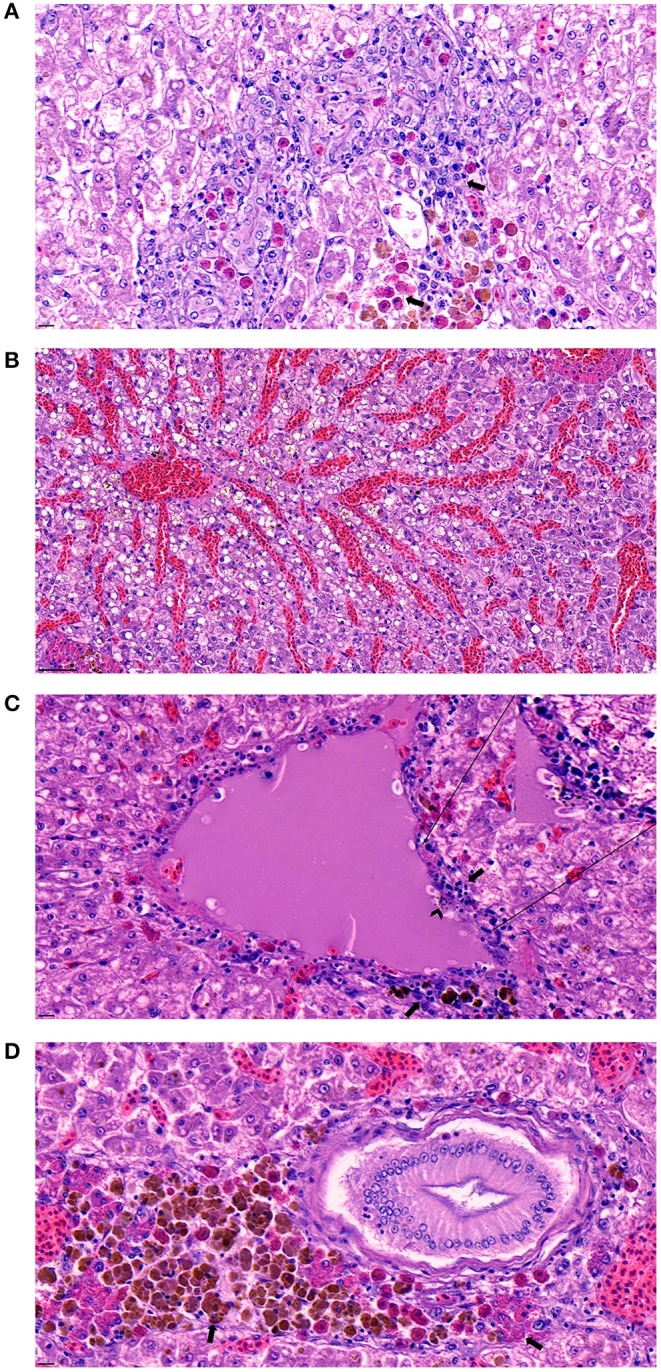
**(A)** Signs of necrotizing areas in liver with cytoplasmic vacuolation accompanied by phagocytized bacterial rods, heterophils, and melanomacrophages (arrow) surrounding the area (Scale bar indicating 10 μm). **(B)** Sever congestion observed in liver of infected fish (Scale bar indicating 50 μm). **(C)** Signs of vasculitis with cellular debris (arrow) accompanied by diverse immunocyte infiltration surrounding the vessel and macrophage phagocytized bacterial rods (arrowhead) detected in the area (Scale bar indicating 10 μm). **(D)** Melanomacrophage and heterophils (arrow) concentrated around a bile duct on the liver (Scale bar indicating 10 μm).

The histopathological examination of the liver, spleen, and kidneys indicated that the bacterial infection of strain SNU2 induced similar pathological changes to that observed in a previous study performed on a Nile tilapia farm in Costa Rica (15). Most of the lesions were concentrated on the liver rather than the spleen or kidneys. The liver showed signs of multi-focal necrotic lesions throughout the entire organ, and severe congestion was occasionally observed (Figures 3A,B). Signs of vasculitis and an infiltration of multiple immunocytes, including macrophages and lymphocytes, were observed; macrophages containing phagocytized bacterial rods were additionally observed (Figure 3C). Moreover, multiple vacuoles were detected throughout the liver. Differences found, relative to past studies, include the signs of vasculitis causing bacteremia and heterophil infiltration, with melanomacrophage observed near bile duct (Figure 3D) (15). The spleen also showed similar signs of vasculitis and macrophage infiltration but the degree of severity was less that the liver. The specific lesions uniquely observed in this study included signs of vasculitis on liver, which may be the cause of bacteremia causing septicemia in fish, and the unusual appearance of concentrated melanomacrophages and heterophils were observed surrounding the bile duct. Because immunocytes, such as macrophages and lymphocytes were clustered around hepatic veins and the splenic artery in the histopathological analysis, we can speculate the intraperitoneally injected bacteria may have infected the blood stream. Therefore, similar pathological lesions observed in past studies, of multi focal necrosis and infiltration of macrophages and lymphocytes, were observed in both the spleen and liver.

### Potential Virulence and Effect of *E. anguillarum* Strain SNU2 in Aquaculture

To our knowledge, this is first report investigating the pathogenicity of *E. anguillarum* isolated from a tilapia farm located in Asia. However, as *E. anguillarum* species were divided latest from *E. tarda*. there may be some possibility that the bacteria was not reported correctly on past research. As tilapia is one of the most consumed fish species in Asia and only few research were done considering the disease distribution of cultivated tilapia in Korea, our aim for this study was to analyze the bacterial strain using phenotypical characterization and histopathological approaches to improve the current understanding and enhance disease prevention strategies.

As this is the first study reporting an *E. anguillarum* infection in a tilapia farm located in Asia, the pathogenicity of the strain was estimated by implementing a challenge trial in tilapia itself, which was different to past studies where such trials were performed in zebrafish (3). Moreover, histopathological analysis was undertaken to investigate the pathogenicity of the strain and compare the results to existing studies. Further investigation into disease prevention and regulation is needed as an alternative to antibiotic treatments, to reduce the economic losses caused by Edwardsiellosis in aquaculture industries. Considering the potential virulence and the widespread distribution of *E. anguillarum* infection, these bacteria may be another global threat to aquaculture industries alongside *E. piscicida*. Most countries in Asia, including China, Japan, and Korea, consume most fresh water fish in raw form; this must be considered, as tilapia are typically grown under substandard conditions owing to its natural resistance and adaptability to various environmental conditions, compared to other fish species like trout and salmon. As *E. tarda* is known as a zoonotic pathogen that infects immunocompromised patients (26), it might be possible for *E. anguillarum* to follow a similar route of pathogenicity; the bacteria was able to grow up to 40°C, indicating that it is capable of growth during human infection.

To conclude, the current study presents the bacterial characterization and histopathological analysis of an *E. anguillarum* strain isolated from a tilapia farm located in Korea. To our knowledge, this is the first report of an *E. anguillarum* infection in tilapia in a farm in Asia that induced mortality and economic losses. The biochemical characterization of the bacteria showed that the strain could not be distinguished from other *Edwardsiella* species using the standard API test. In addition, histopathological analysis showed similar lesions to recent studies performed in tilapia farms located in Costa Rica, however, a few key differences were additionally observed including signs of vasculitis concentrated especially on liver and heterophils infiltration along the hepatic vein which were clearly differed to former studies since histological lesions were concentrated on kidney. The pathogenicity challenge trial showed the strain to be virulent, and a causative agent of edwardsiellosis-induced mortality in tilapia. Further studies regarding the pathogenicity of the bacterium as a zoonotic pathogen and investigation into alternative methods to antibiotic treatment are required for the prevention and control of the disease in aquaculture industries.

## Data Availability Statement

The datasets generated for this study can be found in the GenBank NIH database (https://www.ncbi.nlm.nih.gov/genbank/), using the following accession numbers: MN203720 for 16S rRNA gene data; MN225931, MN225932, MN225933, MN225934, MN225935, MN225936, MN218777 for seven house-keeping genes of strain SNU2.

## Ethics Statement

Ethical review and approval was not required for the study because the Korean Institutional Animal Care and Use Committee doesn't provide ethical approval for commercial fish. The authors guarantee that strict guidelines for handling fish were followed.

## Author Contributions

WO, JJ, and SP designed the study. WO performed the experiments and prepared the manuscript. WO, SG, HK, SWK, SH, SY, SGK, JWK, JK, and JJ analyzed the data. JJ and SP revised the manuscript.

### Conflict of Interest

The authors declare that the research was conducted in the absence of any commercial or financial relationships that could be construed as a potential conflict of interest.
